# A Retrospective Study to Assess the Clinical Benefit of Ultrasound Scans Alone in the Diagnosis of Acute Appendicitis With Normal Biochemical Findings

**DOI:** 10.7759/cureus.73070

**Published:** 2024-11-05

**Authors:** Sima Patel, Ceri Gillett

**Affiliations:** 1 Surgery, New Cross Hospital, Wolverhampton, GBR

**Keywords:** appendicitis, general surgery, investigation, quality improvement project, ultrasound

## Abstract

Introduction

Acute appendicitis can affect patients of any age, although it is uncommon in the extremes of age. Timely diagnosis and management of acute appendicitis can have a significant positive effect on both patient care and the local population's wider health. A variety of different imaging modalities exists to investigate possible appendicitis including ultrasound (US) scan, computed tomography (CT) scan, and magnetic resonance imaging (MRI). However, clinicians are at risk of overreliance on radiology alone, rather than clinical history and examination findings, to make a diagnosis.

Method

A team at a district general hospital (DGH) within the United Kingdom carried out a retrospective, single-centre audit over a three-month period using a general surgical handover list to collate data. The aim of the audit was to assess the value US added when reviewing paediatric patients for suspected appendicitis if biochemical results and clinical findings were within normal parameters.

Results

Following the exclusion of inappropriate patients, 76 patients were incorporated in the data collection process. Of those patients reviewed, eight (11%) of the population had a positive diagnosis of appendicitis, zero (0%) of which had positive US results. Fifty-three patients (70%) had negative US findings, and 23 patients (30%) did not receive a US at all. Fifty-three patients in whom a US was performed (100%) had a negative US as well as normal inflammatory markers (white cell count, C-reactive protein, and neutrophil).

Conclusion

US alone is not sufficient to rule out appendicitis and should be used when there is a moderate to high degree of clinical suspicion of appendicitis. US should be used, in conjunction with clinical and biochemical evidence, to help confirm a diagnosis of appendicitis only.

## Introduction

Acute appendicitis is a common presentation among the general surgical team. It is defined as inflammation of the vermiform appendix, a blind-ended tube found at various locations at the distal end of the caecum [1]. Although acute appendicitis can present at any age, it typically presents between the first and second decades of life. It has a lifetime prevalence of 7%, with a male-to-female ratio of 1:4 and a lifetime risk of 6.7% and 8.7%, respectively [1-3].

Patient history and examination form the foundation of clinical assessment for suspected appendicitis. Murphy first described the classical migratory right iliac fossa pain often seen in appendicitis as having a strong association with inflammation of the appendix. The presence of this clinical finding is a strong diagnostic indicator of acute appendicitis, but as this can be absent in 50% of cases, the lack of it does not rule appendicitis out [1,3,4].

Clinical features

Abdominal pain is described as vague or colicky in the first instance due to midgut parietal peritoneal irritation. This abdominal pain later becomes more localised and sharper in nature as visceral peritoneal irritation occurs due to ongoing inflammation. Other aspects of the history which should raise clinical suspicion of acute appendicitis include nausea, loss of appetite, diarrhoea (suggestive of subcaecal and pelvic appendix due to irritation of the rectum), and urinary symptoms (suggestive of pelvic appendix causing irritation of the bladder) [1,2]. Vomiting may be associated with acute appendicitis if there is a pre-ileal position appendix which causes irritation of the ileum. It is important to note that the presence of vomiting may also represent alternative pathology such as gastroenteritis. Children and infants may present atypically so a high degree of clinical suspicion should be employed in this group of patients [1-4].

Imaging and classification

Acute appendicitis is classified as either simple or complicated, the latter being in the presence of perforation or gangrenous appendix or when an abscess is associated with the inflamed appendix [3]. The diagnosis of acute appendicitis is usually a clinical one, but there are various imaging modalities including ultrasound (US) scan, computed tomography (CT) scan, and magnetic resonance imaging (MRI) that can help support the diagnosis when there is clinical doubt. With increasing access to these scans, clinicians may feel obligated to rely on such methods to inform a diagnosis, even where there is low clinical suspicion of pathology.

Imaging allows clinicians to avoid unnecessary appendectomies while ensuring there is no delay in diagnosis. Compression US was first introduced by Puylaert in 1986, and over time, the technique has been studied and improved and is now considered the first-line imaging of choice in children with suspected appendicitis by the American College of Radiology [5,6]. On US, a positive scan is considered one which has a non-compressible appendix, with a maximal diameter greater than 6 mm and wall thickness greater than 3 mm (with or without the presence of a faecolith). Other clinical features associated are hyperaemia of the appendicular wall in the early stages (although this may not be present when necrosis is present), free fluid or abscess in the pelvis or around the appendix, enlarged lymph nodes, and increased echogenicity of peri-appendiceal fat [1].

Management

Following appropriate resuscitation, the management of appendicitis can be broken down into conservative or surgical. The rate of perforation has been documented to range from 16% to 40%, with a high frequency of perforation noted in younger patients [7]. It is therefore critical that there is early recognition and management in these patients as appendiceal perforation is associated with an increase in morbidity and mortality when compared to the non-perforated appendix.

The diagnosis of appendicitis is a clinical challenge. While scoring systems exist to aid the clinician, these are not a replacement for good patient history and examination, as well as utilising biochemical and radiological support. We looked at the value of US in the diagnosis of acute appendicitis in paediatric patients to assess whether they aided diagnosis when biochemical results were within normal limits and clinical suspicion of appendicitis was low.

## Materials and methods

A single-centre, retrospective snapshot audit was conducted in a district general hospital (DGH) within the National Health Service (NHS) over a three-month period. We used the general surgical handover list, used to record all inpatient referrals, to collate a list of patients who were referred for suspected appendicitis over the assigned time period who met the inclusion and exclusion criteria. Data collection was carried out between January 1, 2024, and March 31, 2024, inclusive.

This study was designed to assess the viability of US alone in the diagnosis of appendicitis in the paediatric population when there were a low clinical suspicion and normal biochemical results. All paediatric patients newly referred to the general surgical team for suspected appendicitis were included in the data collection. Paediatric patients were defined as any patient 18 years of age and under.

We identified 76 paediatric patients who were suitable for data collection over the assigned study period. An exclusion criterion was implemented to maximise the ability to assess the usability of US in the diagnosis of appendicitis in these patients. Those who met the exclusion criteria included those seen as part of a regular review as opposed to being a new referral, as the authors felt this had the ability to skew the data. Patients in which there was another surgical diagnosis being considered were also excluded from the data collection as the authors felt that alternative imaging modalities may be appropriate and therefore skew the data. Those in whom appendicitis has been proven on imaging prior to the referral were excluded, as these patients were triaged for management rather than reviewed and so did not reflect the parameters of this study. All patients over the age of 18 were excluded from the data collection as the focus of this study was to assess the usability of US in the paediatric population. Table 1 highlights the inclusion and exclusion criteria for data collection.

**Table 1:** Inclusion and exclusion criteria for data collection

Inclusion criteria for data collection	Exclusion criteria for data collection
All paediatric patients	Those who were seen as part of a regular review rather than a new referral
All patients referred to the general surgical team for suspected appendicitis within the study period	Those where another surgical diagnosis, other than appendicitis, was being considered
	Those who were over the age of 18
	Those who already had imaging-proven appendicitis prior to the referral

Primary parameters were highlighted to assess whether there was a high clinical suspicion or biochemical evidence to suggest that appendicitis was a leading differential diagnosis. The parameters were determined by the authors as those which would show support for an inflammatory process and therefore help support the diagnosis of appendicitis while ruling out other causes for the patient's symptoms. Patient demographics were recorded to assess the incidence of appendicitis within the local population.

The secondary aim of the study was to assess whether obtaining a US scan delayed ongoing care for the patient. Data collection around the request of US as well as the timing of US was collected to help determine both the viability of the scan and whether this enhanced or compromised patient care. Amylase and bilirubin were included in the data collection due to studies suggesting hyperbilirubinemia, and a rise in amylase, can be associated with acute appendicitis [8,9]. Secondary parameters were established to assess whether investigations led to a delay in management, as such delays may impact both patient health and resource allocation to other patients. The primary and secondary parameters are shown in Table 2.

**Table 2:** Primary and secondary parameters

Primary parameters	Secondary parameters
Patient identification number	How long was an ultrasound performed from the original request?
Patient age	
Patient gender	Was surgery performed during admission?
Ultrasound scan requested	If surgery was performed, did histology confirm acute appendicitis?
Confirmed diagnosis on discharge	
White cell count	
Bilirubin	
Amylase	
Urine dip	
Pregnancy test	

Classification of positive findings

All US were requested post-review of patients by the general surgical team. The general surgical review was performed by either a registrar or a senior house officer. When reviewing US reports, a scan was deemed positive where the report had the following phrases: "findings in keeping with appendicitis", "findings consistent with appendicitis", "appendicitis most probable diagnosis", "scan features highly suggestive of appendicitis", and "history and clinical findings along with US report are consistent with appendicitis". A US report was then deemed negative where the report had the following phrases: "no features of appendicitis", "this study does not account for patient symptoms", "appendix not visualised", and "appendicitis cannot be ruled out on US study". For those who underwent surgical intervention, histology reports were reviewed to assess if histological findings confirmed appendicitis and if these were consistent with both radiological and clinical findings.

Timing of scans

Timing of US requested and performed were reviewed to determine the average length of time taken for US to be performed and reported. The aim of this was to determine whether the use of US aided the diagnosis or caused a delay in patient management and care.

## Results

Following a review of the general surgical acute referrals list, 80 patients were initially highlighted as being appropriate for data collection. Of the 80 patients highlighted, three patients were excluded from the data collection process due to having obtained daily reviews as part of the on-call team, and one patient was excluded due to being outside the study population age group. This generated a list of 76 patients meeting the study inclusion and exclusion criteria and so suitable for data collection.

As well as reviewing the demographics of those referred for suspected appendicitis, we also reviewed their clinical presentation, at the point of referral, to ensure that the clinical diagnosis by the referring team was possible appendicitis. Demographic data, as well as clinical characteristics at presentation, are shown in Table 3 and Table 4 below.

**Table 3:** Characteristic noted at presentation

Parameter assessed	Number of patients (%)
Fever within 24 hours of referral	0 (0%)
Right iliac fossa pain within 24 hours of referral	76 (100%)
Right iliac fossa pain at review by surgeons	8 (11%)
Raised white cell count at the time of referral	14 (18%)

**Table 4:** Demographics of patients in the study

Age group of patients in the study			
0-5 years	6-10 years	11-15 years	16-18 years
3 patients (4%)	27 patients (36%)	31 patients (40%)	15 patients (20%)

**Figure 1:**
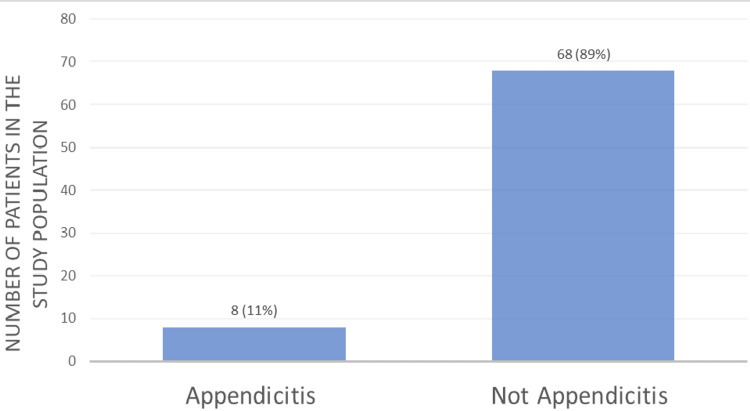
Clinical diagnosis of appendicitis over the three-month study period

The mean age of patients in the study was 13 years, the median age was 12 years, and the age range was from birth to 18 years old. Of the 76 remaining patients, eight patients (11%) had a clinically suspected diagnosis of appendicitis, with six patients (8%) undergoing a diagnostic laparoscopy and appendectomy with subsequent histology displaying positive findings. One patient (1%) who had clinical features of appendicitis (right iliac fossa pain and rise in biochemical markers in keeping with inflammation), as well as radiological findings of appendicitis in the form of CT, was treated conservatively at the patient's request. One patient (1%) was transferred to a tertiary centre and then subsequently treated conservatively for appendicitis with intravenous antibiotics. Figure 1 shows the number of patients diagnosed with appendicitis in the study population over the three-month study period.

**Figure 2:**
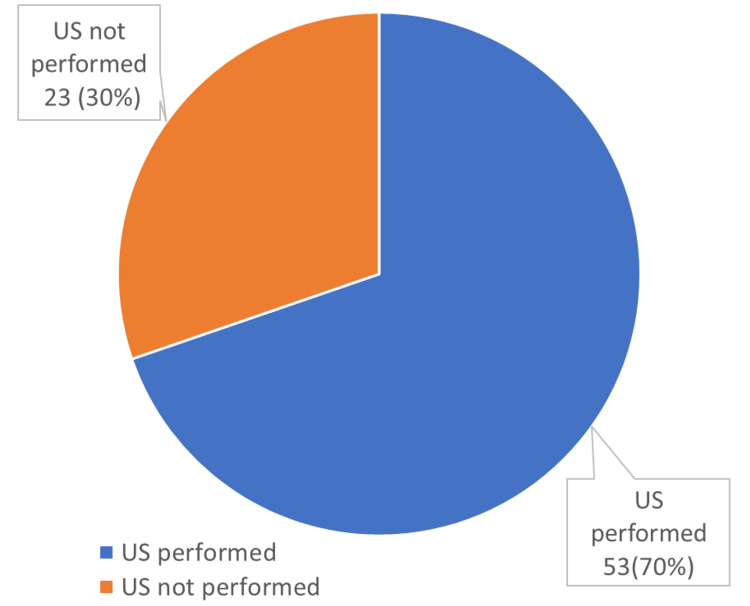
Ultrasound scan use in suspected appendicitis

From the 76 patients reviewed, 53 patients (70%) underwent radiological imaging in the form of a US. Of those who obtained a US, zero (0%) patients had a positive finding of appendicitis. One (1%) of the patients, who had a negative US, was still noted to be symptomatic, and there was a high degree of suspicion for acute appendicitis. As a result, this patient underwent a CT scan, which later confirmed acute appendicitis. Figure 2 shows the use of US in the assessment of possible appendicitis over the three-month study period.

Data showed that clinical findings alone were sufficient to diagnose appendicitis in six cases (8%), and this led to surgical intervention in the form of a diagnostic laparoscopy and appendectomy. US was not obtained in any of these cases prior to surgical intervention. Subsequent histology on these cases confirmed acute appendicitis. One patient (1%) in whom appendicitis was diagnosed, following an abdominal pelvis contrasted CT scan, was treated conservatively at the patient's request. One patient (1%) was noted to be critically unwell at the time of initial review and underwent an urgent transfer to a tertiary centre due to clinical concerns and therefore was not investigated or managed at the primary presenting hospital.

When reviewing patients' biochemical findings in those where a histological diagnosis of appendicitis was made, eight (100%) patients were noted to have a rise in white cell count (WCC), neutrophils, and C-reactive protein (CRP).

Of the remaining 68 patients in which appendicitis was ruled out, 45 patients (59%) had a diagnosis at discharge recorded as "non-specific abdominal pain" with all three inflammatory marker tests (WCC, neutrophils, and CRP) within normal limits. In six patients (8%), inflammatory markers were noted to be raised, but appendicitis was ruled out. Of those six patients (8%) in which appendicitis was excluded, one patient was diagnosed with sickle cell crisis, one patient with cholangitis, one patient with pyelonephritis, and one patient with scarlet fever, while two patients were diagnosed with pancreatitis. In the remaining 17 patients (22%), two patients were diagnosed with constipation, five patients were diagnosed with gastroenteritis, two patients were diagnosed with gynaecology-related pathology, and eight patients were diagnosed with mesenteric adenitis.

**Figure 3:**
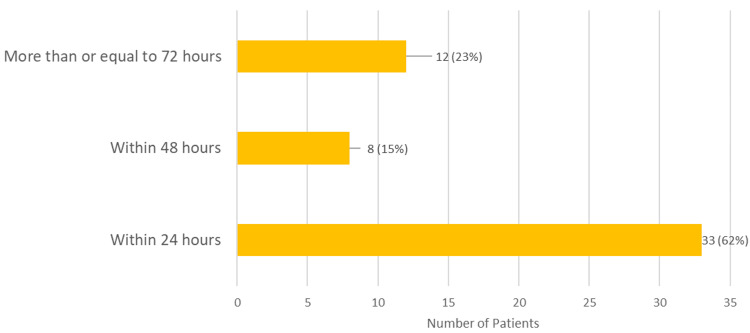
Time frame of ultrasound scans performed

The data collected showed that in 53 cases (100%) where the US was negative for appendicitis, WCC, neutrophil, and CRP were within normal range. Patients who received a negative diagnosis of acute appendicitis were assessed for the re-presentation of symptoms which would have suggested a missed diagnosis of appendicitis. Zero patients (0%) re-presented within the three-month review period. Data was also assessed for the time taken to obtain a US. While 41 (77%) of scans were obtained within a 48-hour window, 12 (23%) scans took over 72 hours to be completed and reported. Of those, zero (0%) were positive for appendicitis. Figure 3 shows the time frame of scans performed.

Seventy-six patients (100%) in the study had urine dips performed as part of the initial bedside investigation. Of those patients who had a positive finding for appendicitis, zero patients (0%) had a urine dip positive for nitrates, leucocytes, protein, blood, or ketones.

We looked at serum amylase and bilirubin as secondary parameters to assess if a rise in these levels was associated with perforated appendicitis. Zero (0%) of patients where a positive diagnosis of appendicitis was made, and in whom serum amylase had been performed, were noted to have a rise in these levels. However, four out of the eight patients in which appendicitis was diagnosed (50%) did not have amylase checked as part of the initial biochemical workup. Seven patients out of eight patients (87%) in which a positive diagnosis of appendicitis was made had serum bilirubin levels checked as part of their initial investigations, of which zero (0%) were noted to have a rise in their bilirubin. The number of patients diagnosed with acute appendicitis over the three-month study period was eight patients (11%), with a male-to-female ratio of 3:1.

## Discussion

The appendix is a narrow tube of variable length, typically between 6 and 10 cm long, which is attached to the distal end of the caecum [1]. The position of the appendix can be variable and include retrocaecal, retrocolic, pre-ileal, post-ileal, subcaecal, and pelvic. Appendicitis is the inflammation of this blind-ended tube. The clinical causes of appendicitis are variable and are related to the blockage of and subsequent inflammation of this tube. Blockage and subsequent inflammation of the appendix can be caused by the following: lymphoid tissue hyperplasia, impacted stool, foreign body, parasites, and in rare instances malignancy [10,11]. Luminal obstruction leads to increased mucus secretion into the lumen, where this increased mucus in a blocked tube provides a breeding ground for bacterial overgrowth. This bacterial overgrowth can then lead to inflammation, reducing the lymphatic and venous drainage from the appendix leading to subsequent ischaemia, necrosis, and potential perforation [10,12].

Acute appendicitis remains one of the most common surgical emergencies encountered by the general surgeon, with around 50,000 acute appendectomies carried out annually in the United Kingdom [11]. The incidence of appendicitis or appendectomies in Western Europe, from 2000 onwards, is quoted to be 151 per 100,000 person-years [6,12]. The incidence of appendicitis in these countries has become more stable; however, a fall in the number of appendectomies has been noted [13]. This fall could be a result of improved modalities of investigation, the development of medical conservative management options, as well as patients engaging in healthcare and vocalising their choice of management. All the above means fewer operations are being undertaken [14].

Acute appendicitis can present within any age group, but prevalence was noted to be highest within the 10-20-year-old age group. Variations in incidences related to sex, ethnicity, age, and obesity as well as seasonal variations [7,15]. The higher prevalence in this age group is likely due to increased lymphoid follicular hyperplasia [3].

Imaging modality

First established in 1986, graded compression US continue to be utilised in the diagnosis of acute appendicitis. As well as being non-invasive and readily available and avoiding the use of ionising radiation, US has well-defined direct and indirect signs which correlate to a positive diagnosis of acute appendicitis [16]. The major drawbacks of US are that they are user-dependent and sensitivity is limited, especially when compared to other modalities of investigation such as CT and MRI. Artefacts such as bowel gas obscuring potential positive findings may also affect the sensitivity of US. The accuracy of a US is thought to be between 71% and 97%. CT have a higher accuracy (between 93% and 98%); however, the use of ionising radiation, complications from the use of contrast media, and costs limit its use, especially among the paediatric population [16,17]. The average radiation exposure level of an abdominal CT is only slightly above background radiation at 4 mSv and 3 mSv, respectively. It is estimated that the lifetime risk of developing a malignancy due to radiation exposure, if an abdominal CT is performed, is 0.18% for a one-year-old child and 0.11% for a 15-year-old child at a standard radiation dose [15,17,18]. However, it is worth noting that low-dose CT can be used in practice with a similar sensitivity to US and normal-dose CT [15,18]. The accuracy of MRI is thought to be 97%, so is comparable to CT; however, MRI are associated with higher costs, and the interpretation of images generated requires expert input [18].

The study highlighted 80 potentially suitable patients for inclusion; however, four (5%) of these were excluded due to not meeting the pre-defined inclusion criteria. Of the remaining 76 patients, appendicitis was positively diagnosed in 11% of the population with a male-to-female prevalence of 3:1. Six patients (8%), who had a positive diagnosis of appendicitis, were diagnosed on history, examination, and biochemical results. Of the remaining 68 patients, 53 (78%) underwent US which led to a negative diagnosis of appendicitis.

The National Cancer Institute, the American College of Radiology, and the American Academy of Pediatrics recommend US as the first-line imaging modality of choice when assessing paediatric patients for acute appendicitis. It is important to consider both availability and inconsistency in the result of the scan when interpreting results. Variations in scan results can be due to varying body habitus, the skill level of the sonography, and potential artefacts obscuring views [6,19]. Studies have also concluded that factors such as sonographers overlooking an inflamed appendix, visualising the appendix but not appreciating that it is inflamed, or concluding the symptoms are due to another pathology lead to false-negative US results [16]. Studies have shown that when a negative US is generated, but there is a high clinical suspicion of appendicitis, reassessment of the patient should be undertaken with the consideration of repeat imaging or alternative investigation modalities [16].

The sensitivity rate of US is between 71% and 94% with a large variation due to the aforementioned reasons. The specificity rate of US is between 81% and 98% [18]. Data in this retrospective study showed a lower sensitivity rate than those quoted for a multitude of reasons including sonographer skill level variability, limited number of available sonographers due to national holidays, and wide variations in body habitus of the paediatric population. US have a positive likelihood ratio between 6 and 46 and a negative likelihood ratio between 0.08 and 0.30. Therefore, while able to confirm appendicitis, they are unable to rule out appendicitis. A retrospective study showed that US had a high specificity and negative predictive value when attempting to exclude acute appendicitis or faecolith [18]. A review of data from this study suggested that US were being used to rule out appendicitis, even when there was a low clinical suspicion of appendicitis. Given the positive and negative likelihood ratios quoted, clinical value is limited when trying to rule out acute appendicitis. The data from this retrospective audit also showed that when biochemical markers were within normal limits, US was negative for appendicitis with zero (0%) of these patients re-presenting for review within the three-month study period.

The primary aim of this audit was to assess whether US aided the management of patients where appendicitis was the primary clinical suspicion. The secondary aim of the study was to assess whether obtaining a US scan delayed ongoing care for the patient. The data showed that while 33 (62%) of US scans were performed within 24 hours, 20 (38%) of scans were performed between 48 and 72 hours. This showed that for 38% of patients, there were potential delays in diagnosis and management, with management ranging from surgical intervention to reassurance and discharge from the service. The morbidity and mortality are associated with the stage of the disease and increase in the case of perforation [1]. Perforation is more common in the elderly and young and with it comes an increase in mortality at 5.1 per 1000 [1,20]. This increase in mortality is why early diagnosis and management are vital to ensure good patient outcomes [1].

The inappropriate use of US wastes resources in the form of both equipment use and allocation and clinician time. The inappropriate use of US also affects financial resource distribution with the avoidance of inappropriate scanning leading to resources being allocated elsewhere. Evidence suggests that US is recommended for children and pregnant patients. However, patient selection is key, and these scans should not be used to rule out appendicitis but rather confirm appendicitis when there is a high degree of clinical suspicion.

A randomised controlled study, carried out among 600 patients in the United States, evaluated the use of US against CT in a paediatric population. The results showed that while US had a diagnostic accuracy of 91%, when there was diagnostic uncertainty, the use of CT was also beneficial in the management of this population group [19]. Within the NHS in the United Kingdom, rates of US performed vary among different areas. Nationally, 1,347 US are performed per 10,000 people [16]. North of the country showed a high concentration of US; however, the number of scans performed is not affected much by age in comparison to other imaging modalities such as CT [17].

Biochemical results

Study data showed that 100% of patients who had a positive diagnosis of appendicitis were noted to have a rise in WCC, neutrophils, and CRP. In isolation, a rise in inflammatory markers is not specific to acute appendicitis and lacks accuracy for the diagnosis of acute appendicitis [15,18]. However, appendicitis requires an inflammatory process to occur so a rise in these inflammatory markers combined with clinical examination findings, with the aid of potential scoring systems or imaging, can prove helpful in the diagnosis of acute appendicitis. It is worth noting that although an inflammatory response is the pathophysiology of appendicitis, a study of 845 individuals, with a median age of 11, found that 20% of patients had appendicitis with a WCC less than 10×109/L [15]. The World Society of Emergency Surgery updated the Jerusalem guidelines in the management of acute appendicitis, by recommending the incorporation of both biochemical results and scoring systems to help determine if further imaging is required in the assessment of paediatric patients with acute appendicitis [15].

Other biochemical markers such as CRP, calcitonin, and calprotectin, as well as the APPY1 biomarker panel which combines CRP, WCC, and myeloid reactive protein level, can be used to rule out or diagnose appendicitis. Studies show that in children, when APPY1 is utilised alone, it has a 98% sensitivity and when combined with US it has a 99% sensitivity. This means that a normal test missed 1-2% of patients with appendicitis [16-18].

Scoring system

There are several different scoring systems that can be used to help aid the diagnosis of acute appendicitis. More commonly used scoring systems include the Alvarado Score, Paediatric Appendicitis Score, and Appendicitis Inflammatory Response Score (see Appendices).

The scoring systems categorise patients into low-, moderate-, and high-risk groups, which can then help guide the further management of the patients and determine if further imaging is required. The Alvarado Score has been widely studied and tested and is suitable for use in both the paediatric and adult populations. The Paediatric Appendicitis Score incorporates similar features as the Alvarado Score but also incorporates additional signs, which may be more prevalent in the paediatric population. In children, it has been shown that the Paediatric Appendicitis Score outperforms the Alvarado Score. The Appendicitis Inflammatory Response Score uses fewer signs when compared to the Alvarado Score and the Paediatric Appendicitis Score; however, it does use biochemical markers to help guide severity. None of the aforementioned scoring systems rely on imaging but rather are used to assess if further imaging is required [15,18].

Data from this study highlighted that no scoring system was used to help stratify risk to the population group. Studies have shown that the use of scoring systems can help determine risk as well as the requirement for imaging in patients with suspected appendicitis [1,2,15,16].

Limitations of the study

This study was performed as a snapshot, and therefore, the population size is limited. To further enhance the study conclusion, the authors aim to repeat the study over a longer time period of time to ensure a larger sample size. The study was carried out at a single DGH and so results may only reflect the position locally rather than nationally. This would be overcome by incorporating other hospitals in future studies, with similar demographics and resource availability across England.

The hospital in which this retrospective audit was carried out only had a limited number of appointments for US with all scans being performed between 0900 and 1700 Monday to Sunday. Further to this, the study was performed over a three-month period which included public holidays. This means the number of staff was limited due to both public holidays and additional annual leave taken by sonographers for various reasons including the need for childcare associated with children's school holidays.

In combination with fewer sonographers working, within the DGH the study was carried out at, US performed in a paediatric patient requires a specialist sonographer, meaning appointment slots were further limited. These would have affected both how much time it took to obtain a US and ultimately time for diagnosis. To overcome this, a follow-up study would be carried out over a 12-month study period to get a broader overview.

## Conclusions

Acute appendicitis is a common presentation to the general surgical team and so efficient diagnosis and management are vital. 

This study highlights that US are unlikely to be of significant clinical benefit when clinical and biochemical findings do not support a diagnosis of appendicitis. US should be used, in conjunction with clinical and biochemical evidence, to help confirm a diagnosis of appendicitis only. The use of US alone is not sufficient to rule out appendicitis and dooms the clinician to failure. The misuse of US can lead to delayed patient care and inappropriate resource allocation, which can have a significant effect on the wider healthcare population.

## Appendices

**Table 5:** The Alvarado Score

Signs/symptoms	Points
Migratory abdominal pain	1
Anorexia	1
Nausea/vomiting	1
Rebound tenderness	1
Temperature ≥37.3°C	1
Polymorphonucleocytes ≥75%	1
White cell count ≥10×10^9^/L	2
Right lower quadrant pain	2
Total possible score	10

**Table 6:** The Paediatric Appendicitis Score

Signs/symptoms	Points
Migratory abdominal pain	1
Anorexia	1
Nausea/vomiting	1
Temperature ≥38°C	1
White cell count ≥10×10^9^/L	1
Polymorphonucleocytes ≥75%	1
Rebound tenderness	2
Right lower quadrant pain	2
Right lower quadrant pain with coughing/hoping/percussion	2
Total possible score	12

**Table 7:** The Appendicitis Inflammatory Response Score

Signs/symptoms	Points
Vomiting	1
Right iliac fossa pain	1
Temperature ≥38.5°C	1
White cell count ≥10-14×10^9^/L	1
White cell count ≥15×10^9^/L	2
Polymorphonucleocytes ≥70-80%	1
Polymorphonucleocytes ≥85%	2
C-reactive protein 10-49 g/L	1
C-reactive protein ≥50 g/L	2
Rebound tenderness: light	1
Rebound tenderness: medium	2
Rebound tenderness: strong	3
Total possible score	12
